# Pediatric neurodevelopment by prenatal Zika virus exposure: a cross-sectional study of the Microcephaly Epidemic Research Group Cohort

**DOI:** 10.1186/s12887-020-02331-2

**Published:** 2020-10-10

**Authors:** Paula Fabiana Sobral da Silva, Sophie Helena Eickmann, Ricardo Arraes de Alencar Ximenes, Ulisses Ramos Montarroyos, Marilia de Carvalho Lima, Celina M. Turchi Martelli, Thalia Velho Barreto de Araújo, Elizabeth B. Brickley, Laura Cunha Rodrigues, Fabiana Cristina Lima da Silva Pastich Gonçalves, Maria Durce Costa Gomes Carvalho, Wayner Vieira de Souza, Demócrito de Barros Miranda-Filho

**Affiliations:** 1 Hospital Universitário Oswaldo Cruz, Recife, Brasil; 2 Universidade Federal de Pernambuco, Recife, Brazil; 3 Instituto de Pesquisa Aggeu Magalhães, Recife, Brazil; 4 London School, London, England

**Keywords:** Child behavior, Child development, Congenital Zika syndrome, Microcephaly

## Abstract

**Background:**

The implications of congenital Zika Virus (ZIKV) infections for pediatric neurodevelopment and behavior remain inadequately studied. The aim of this study is to investigate patterns of neurodevelopment and behavior in groups of children with differening severities of ZIKV-related microcephaly and children with prenatal ZIKV exposure in the absence of microcephaly.

**Methods:**

We conducted a cross-sectional study, nested in a cohort, of 274 children (aged 10–45 months) who were born during the peak and decline of the microcephaly epidemic in Northeast Brazil. Participants were evaluated between February 2017 and August 2019 at two tertiary care hospitals in Recife, Pernambuco, Brazil. We analyzed the children in four groups assigned based on clinical and laboratory criteria: Group 1 had severe microcephaly; Group 2 had moderate microcephaly; Group 3 had prenatal ZIKVexposure confirmed by maternal RT-PCR testing but no microcephaly; and Group 4 was a neurotypical control group. Groups were evaluated clinically for neurological abnormalities and compared using the Survey of Wellbeing of Young Children (SWYC), a neurodevelopment and behavior screening instrument validated for use in Brazil. Children with severe delays underwent further evaluation with an adapted version of the SWYC.

**Results:**

Based on the SWYC screening, we observed differences between the groups for developmental milestones but not behavior. Among the 114 children with severe microcephaly of whom 98.2% presented with neurological abnormalities, 99.1% were ‘at risk of development delay’ according to the SWYC instrument. Among the 20 children with moderate microcephaly of whom 60% presented with neurological abnormalities, 65% were ‘at risk of development delay’. For children without microcephaly, the percentages found to be ‘at risk of developmental delay’ were markedly lower and did not differ by prenatal ZIKV exposure status: Group 3 (N = 94), 13.8%; Group 4 (N = 46), 21.7%.

**Conclusions:**

Among children with prenatal ZIKV exposure, we found a gradient of risk of development delay according to head circumference. Children with severe microcephaly were at highest risk for delays, while normocephalic ZIKV-exposed children had similar risks to unexposed control children. We propose that ZIKV-exposed children should undergo first-line screening for neurodevelopment and behavior using the SWYC instrument. Early assessment and follow-up will enable at-risk children to be referred to a more comprehensive developmental evaluation and to multidisciplinary care management.

## Background

Zika virus (ZIKV) is a teratogenic arthropod-borne flavivirus, and its vertical transmission can lead to fetal injury, which can clinically manifest in a pattern of signs and symptoms recognized as Congenital Zika Syndrome (CZS) [1]. The CZS phenotype is marked by structural defects, including morphological alterations to the limbs, eyes, and brain as well as functional impairments, such as difficulty in swallowing and communication [2].

Current evidence suggests that many of the functional disabilities associated with CZS arise from damage to the developing nervous system. Intrauterine ZIKV infections exhibit a marked neurotropism, and recent studies indicate that ZIKV is able to impair the viability and growth of neural progenitor cells and post migratory neurons  [3–5]. Indeed, microcephaly - a hallmark of CZS - is thought to arise when prenatal infections with ZIKV trigger fetal brain disruption sequence, resulting in brain volume loss, reduced intracranial pressure, and skull collapse  [2, 6].

Despite an increasing understanding of the pathogenic mechanisms of fetal ZIKV infection and the resultant structural changes, the long-term implications of congenital ZIKV exposure on neurodevelopment and behavior remain understudied. To address the pressing need for research on the prognosis of children exposed to ZIKV during pregnancy, we evaluated children enrolled in the Cohort of Children of the Microcephaly Epidemic Research Group (MERG) using the Survey of Wellbeing of Young Children (SWYC) screening instruments  [7–9], which are validated for use in Brazil  [10]. Although there are several instruments available for identifying signs of risk for developmental delays,  [11] the SWYC is particularly advantageous due to its ease of use as a first-line screening tool and its capacity to assess children with varying degrees of neurological impairment.

The aim of this study is to investigate the neurodevelopment and behavior of groups of children with severe and moderate ZIKV-related microcephaly and children with prenatal ZIKV exposure in the absence of microcephaly, born between 2015 and 2017 in Pernambuco State, the epicenter of the microcephaly epidemic in Northeast Brazil  [12].

## Methods

We conducted a cross-sectional study, nested in the MERG pediatric cohort of children born during the peak and decline of the ZIKV-related microcephaly epidemic in the Northeast of Brazil. Detailed clinical histories for all participating children were available in the core cohort dataset.

The MERG Pediatric cohort includes children recruited from: (i) outpatient care at three health centres who were identified in the peak of the microcephaly epidemic as having a head circumference below 33 cm and/or with severe neurological abnormalities; (ii) the MERG Microcephaly case-control study; and (iii) the offspring of the MERG cohort study of pregnant women with rash. Ethical approval for the recruitment, follow-up, and screening was provided by local institutional review boards (MERG Pediatric Cohort, CAAE 52803316.8.0000.5192; MERG Pregnant Women Cohort, CAAE 53240816.4.0000.5190; MERG Case-Controlled Study 51849215.9.0000.5190). Participants were evaluated between February 2017 and August 2019 at two tertiary care hospitals (the Hospital Universitário Oswaldo Cruz and at the Rehabilitation Center of the Fundação Altino Ventura) in Recife, Pernambuco, Brazil.

**Fig. 1:**
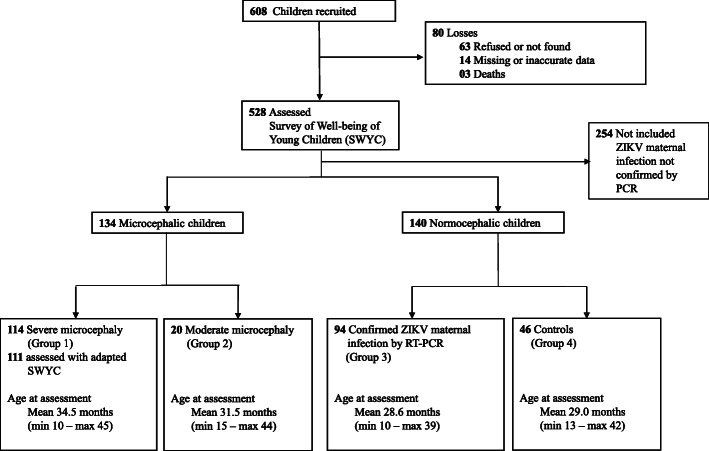
Flow diagram of children enrollment

From a total of 608 children followed in the MERG Pediatric Cohort, SWYC screening information was unavailable in 80 participants who either could not be assessed by SWYC or had inconsistencies in completing the form. Of the remaining 528 children, a further 254 normocephalic children were not included as their mothers had no laboratory testing for ZIKV by PCR. Thus, the analysis was conducted on the 274 children who completed neurodevelopmental screening and whose mothers had their ZIKV infection status molecularly confirmed during pregnancy. Of note, we restricted inclusion in this study to participants whose mothers underwent PCR testing for ZIKV, as PCR provides the most robust laboratory evidence of an acute infection during pregnancy (Fig. 1).

Group allocation depended on children’s head circumference (HC) and prenatal ZIKV exposure status. To differentiate the severity of microcephaly between groups 1 and 2, we considered the HC measure collected closest to the date of the SWYC assessment. To differentiate between the children without microcephaly in groups 3 and 4, we considered the maternal ZIKV infection testing results obtained during pregnancy and at birth.

The children in Groups 1 and 2 were born during the peak of the microcephaly epidemic and referred for further neuro-pediatric evaluation as part of their tertiary health care for CZS. Group 1 consisted of 114 children with severe microcephaly and other clinical and/or radiological abnormalities consistent with CZS. Severe microcephaly was defined as a HC corresponding to ≥ 3 standard deviations (SD) below the mean for sex and age (corrected for prematurity), according to Intergrowth charts  [13]. Group 2 included 20 children with moderate microcephaly and other clinical or radiological abnormalities consistent with CZS. Moderate microcephaly was defined as a HC between 2 and < 3 SD below the mean [13].

Group 3 included 94 children without microcephaly born to mothers who tested positive for ZIKV during pregnancy by one-step reverse-transcription polymerase chain reaction (RT-PCR) using primers and probes previously described by Lanciotti and et al., [14]. Detailed descriptions of the laboratory testing during pregnancy have been previously published  [15]. Group 4 included 46 neurotypical children with no microcephaly nor any other brain abnormalities detectable by brain ultrasound at birth who were born to mothers with no laboratory evidence of ZIKV infection during pregnancy. The children in Group 4 were recruited at birth as the control group of a case-control study of microcephaly, whose detailed descriptions of ZIKV testing and clinical evaluations have been previously published  [16].

## Screening assessment and clinical evaluations

Trained health professionals evaluated children using the SWYC, a standardized instrument for surveillance of children’s neurodevelopment and behavior, which has been translated into Brazilian Portuguese and validated for use in Brazil [10]. If a child was administered the SWYC more than once during follow-up, we analyzed only the results from the oldest age at screening. Similarly, in the case of repeated neurological evaluations, we used the data from the evaluation that was closest in time to the SWYC assessment.

The SWYC includes: (a) milestones for screening cognitive, language, and motor development in children under 60 months of age,  [7] (b) the Baby Pediatric Symptom Checklist (BPSC) for social/emotional screening of children under 18 months of age [8] and (c) the Preschool Pediatric Symptom Checklist (PPSC) for social/emotional screening of children aged 18 to 60 months  [9] .

For the milestones, each form is age-specific (with corrected age for children born prematurely) and includes 10 items. Children were considered at “risk of developmental delay” when their total score for the milestones fell below the cut-off points for the respective age groups, using the standardized scoring thresholds for both the original test from the United States of America (USA) [7] and the Brazilian version [8]. For the behavioral evaluations, children were considered to have ‘suspected behavior abnormalities’ if they scored ≥ 3 on any domains of the BPSC (i.e., irritability, inflexibility, and difficulty with routines) or ≥ 9 for the PPSC, using the American standardized scoring [9, 10]. Parents were informed of the screening outcomes and received guidance on appropriate home stimulation.

Following initial observations that many of the children with microcephaly did not perform any skills predicted by their chronological age, we adapted the SWYC milestones form to explore the extent of the delays. Specifically, we listed the SWYC milestones from 1 to 36 months of age in increasing order of complexity and evaluated children’s abilities to perform the ranked skills. Assessment using the adapted form was brought to an end for a given child after six answers of “not yet.”

Neurologic assessments were performed by qualified pediatric neurologists. Neurological impairment was identified as the occurrence of abnormalities in consciousness, irritability, tone, muscular trophism, muscular strength, and/or proprioceptive reflexes. Radiological abnormalities were detected by brain ultrasound, computed tomography (CT), and/or magnetic resonance imaging (MRI) and included the presence of calcifications, ventriculomegaly, cortical or cerebellar atrophy, and/or neuronal migration disorder.

## Data management and statistical analysis

We used the chi-squared test to compare the distribution of participants’ characteristics (sex, gestational age, birthweight, small-for-gestational age, neurologic abnormalities identified by clinical evaluation, and neuroimaging abnormalities) across groups 1 to 4. We reported the overall statistical significance (*p*-value for Σ^2 ^test), but also calculated the residuals to identify the cells making the greatest contribution to significant results. We calculated the Chi-squared test for trend to compare the proportions of children ‘at risk’ for neurodevelopmental delays and behavioral symptoms across the four groups.

To evaluate skill performance by age in children with severe microcephaly (Group 1), we applied the adapted milestones form in two age subsets: children evaluated at 24 months of age and younger versus children older than 24 months. The non-parametric Mann-Whitney U test was used to test for differences in the performance of development milestones, measured as ordinal scores on the SWYC adapted form. This test is an alternative to the one-way analysis of variance for data that is not normally distributed.

We considered significant level set up at p < 0.05, and all testing was two-tailed. Data were double entered into the secure digital platform. Analyses were performed using Stata, version 15 statistical software (StataCorp LP) and R software.

A total of 274 infants underwent neurodevelopmental and behavioral assessment. As this is the first study to use SWYC assessment in children with CZS, there are no previous data to inform the sample size calculations. Therefore, we enrolled all children who fulfilled the inclusion criteria.

## Results

The study sample comprised 274 children evaluated using SWYC at a mean age of 31.4 months.

The HC of the children with microcephaly (Groups 1 and 2) ranged from − 2.03 to -10 SD below the mean for age and sex.

Table 1 shows that severe microcephaly cases (Group 1) had a statistically significant higher frequency of abnormal neurological findings (112 of 114 [98.2%]) than the other groups. Similarly, neuroradiological alterations (103 of 106 [97%]) were elevated in Group 1 as compared to moderate microcephaly cases (Group 2) (6 of 16 [37.5%]) and normocephalic children born to ZIKV-positive mothers (Group 3) (5 of 43 [11.6%]). Other clinical findings in Group 1 included: pyramidal syndrome (i.e., defined by the presence of hypertonia, clonus, hyperreflexia, and increased archaic reflexes) (89.2%), inadequate responses to visual stimuli (57%), and inadequate responses to auditory stimuli (14%). The frequency of seizures differed across the ZIKV-exposed groups and were reported 67.9% [72 of 106] of the children in Group 1, 17.6% [3 of 17] in Group 2, and 2.2% [2 of 90] in Group 3 (data not shown). The number of children in the groups varied by outcome as not all of the children were able to participate in all of the assessments.

**Table 1:** Birth characteristics, neurological and radiological characteristics of groups of infants with severe and moderate Zika related microcephaly, as well as those exposed and neurotypical controls

Biological and radiological characteristics	Study Groups				
	**Group 1**	**Group 2**	**Group 3**	**Group 4﻿**	
	**Severe Microcephaly** (*n* = 114)	**Moderate microcephaly** (*n* = 20)	**ZIKV maternal** **infection**^**a**^ (*n* = 94)	**Controls**^**b**^ (*n* = 46)	***P*** **Value**
**Sex, No. (%)**					
Female	64 (56.1)	12 (60.0)	47 (50.0)	25 (54.3)	.*786*
Male	50 (43.9)	8 (40.0)	47 (50.0)	21 (45.7)	
**Prematurity, Weeks of Gestational Age, No. (%)**					
< 37	16 (14.2)	4 (25.0)	6 (7.9)	7 (15.2)	.*249*
≥ 37	97 (85.8)	12 (75.0)	70 (92.1)	39 (84.8)	
* Missing*	1	4	18	0	
**Birthweight, g, No. (%)**					
< 2500	28 (25.5)	10 (58.8)^c^	5 (7.1)	2 (4.3)	*< 0.001*
2500–2999	47 (42.7)	3 (17.6)	14 (20.0)	12 (26.1)	
≥ 3000	35 (31.8)			32 (69.6)	
* Missing*	4	3	4 (23.5)	51 (72.9)	
**Small-for-gestational age, No. (%)**					
Yes	29 (26.4)	8 (47.1)^c^	5 (7.1)	2 (4.3)	*< 0.001*
No	81 (73.6)	9 (52.9)	65 (92.9)	44 (95.7)	
* Missing*	4	3	24	0	
**Clinical neurological abnormalities, No. (%)**					
Yes	112 (98.2)^c^	12 (60.0)	10 (11.1)	3 (7.5)	*< 0.001*
No	2 (1.8)	8 (40.0)	80 (88.9)	37 (92.5)	
* Missing*	0	0	4	6	
**Neuroimaging abnormalities, No. (%)**					
Yes	103 (97.2)^c^	6 (37.5)	5 (11.6)	0 (0)	*< 0.001*
No	3 (2.8)	10 (62.5)	38 (88.4)^d^	46 (100)	
* Missing*	8	4	51	0	

The number of children varied due to missing values

^a^Normocephalic children born to mothers ZIKV PCR+

^b^Normocephalic children, considered neurotypical controls

^c^Cells making the greatest contribution to significant results

^d^For Group 3 brain ultrasound was performed within the first 6 months of life. For children who had the first evaluation over 6 months, neuroimaging was performed only according to clinical indication

For Group 1, SWYC screening indicated risks of development delay in 99% of the children using the Brazilian threshold (100% using the American threshold). Strikingly, none of the children in Group 1 were able to perform the milestone skills expected for their chronological age. For Group 2, SWYC screening indicated risks of developmental delay in 65% of the children using the Brazilian threshold (70% using the American threshold). Of note, Group 3 (ZIKV-exposed children without microcephaly) and Group 4 (neurotypical controls) had similar frequencies of children at risk of development delay. Overall, there was a clear gradient of risk (chi-squared test for trend, df = 3, *p* < 0.01), with a greater proportion of children at risk of developmental delay in the microcephalic groups compared to the normocephalic groups. Results of the behavior evaluations with the BPSC and PPSC are presented together (i.e., independently of age group) due to the small number of children evaluated under 18 months of age. Based on the BPSC and PPSC, there were no significant differences between the four groups in the frequency of children with ‘at risk’ behavior (Table 2).

**Table 2:** SWYC neurodevelopmental and behavioral outcomes in children with severe and moderate Zika related microcephaly, children exposed without microcephaly and neurotypical controls

SWYC components	Study Groups				*P* Value (Σ^2^)
	**Severe** **Microcephaly**	**Moderate microcephaly**	**ZIKV maternal infection**^**a**^	**Controls**^**b**^	
	(*n* = 114)	(*n* = 20)	(*n* = 94)	(*n* = 46)	
**Development milestone (Brazil), No. (%)**					
“At risk”/Needs review	113 (99.1)^c^	13 (65.0)^c^	13 (13.8)	10 (21.7)	< 0.001
“Appears to Meet Age Expectations”	1 (0.9)	7 (35.0)	81 (86.2)	36 (78.3)	
**Development milestone (USA), No. (%)**					
“At risk”/Needs review	114 (100.0)^c^	14 (70.0)^c^	19 (20.2)	12 (26.1)	< 0.001
“Appears to Meet Age Expectations”	0 (0.0)	6 (30.0)	75 (78.8)	34 (73.9)	
**Baby and Preschool Pediatric** **Symptoms Checklist, No. (%)**					
“At risk”/Needs further evaluation or investigation	55 (51.0)	13 (65.0)	40 (42.5)	28 (63.7)	0.70
Adequate	53 (49.0)	7 (35.0)	54 (57.5)	16 (36.3)	
* Missing*	6	0	0	2	

^a^Normocephalic children born to mothers ZIKV PCR+

^b^Normocephalic children, considered neurotypical controls

^c^Cells making the greatest contribution to significant results

Σ^2^test for trend: 151.6 (p < 0.0000001) OR: 1 (controls); 0.58 (ZIKV maternal infection); 6.69 (moderate microcephaly); 406.8 (severe microcephaly)

Σ^2^test for trend: 138.2 (p < 0.0000001) OR: 1 (controls); 0.72 (ZIKV maternal infection); 6.61 (moderate microcephaly); not calculated (severe microcephaly)

Among the Group 1 children assessed for developmental milestones using the adapted SWYC milestone form, 73% (82 of 111) were evaluated after two years of age. Almost all children scored lower than expected, independent of the age of assessment. There were no statistically significant difference in scores by age of assessment except for the milestone of “Laughs” (Table 3).

**Table 3:** Differences in development milestones gains between children with severe Zika-related Microcephaly, divided by age groups, using the SWYC adapted form

Development Milestones	≤ 24 months (*n* = 29)				> 24 months (*n* = 82)				
	Not yet	Some what	Very much	Mean Rank	Not yet	Some what	Very much	Mean Rank	*P* Value^a^
Makes sounds that let you know he or she is happy or upset	0	6	23	53.5	5	6	71	56.9	0.44
Seems happy to see you	1	2	26	54.8	2	4	76	56.4	0.61
Follows a moving toy with his or her eyes	5	10	14	50.3	7	26	49	58.0	0.21
Turns head to find the person who is talking	0	9	20	53.5	5	14	63	56.9	0.53
Holds head steady when being pulled up to a sitting position	9	12	8	54.3	25	30	27	56.6	0.73
Brings hands together	14	6	9	53.9	40	8	34	56.7	0.66
Laughs	1	5	23	50.2	3	2	77	58.0	0.03
Keeps head steady when held in a sitting position	9	14	6	50.3	19	37	26	58.0	0.23
Makes sounds like “ga,“ “ma,“ or “ba”	19	4	6	51.0	46	8	28	57.8	0.27
Looks when you call his or her name	3	9	17	50.3	7	15	60	58.0	0.17
Rolls over	16	8	5	52.4	42	16	24	57.3	0.45
Passes a toy from one hand to the other	26	0	3	52.9	67	4	11	57.1	0.35
Looks for you or another caregiver when upset	7	8	14	56.2	30	6	46	55.9	0.96
Holds two objects and bangs them together	26	1	2	54.7	71	3	8	56.5	0.66
Holds up arms to be picked up	26	2	1	50.8	64	4	14	57.8	0.14
Gets into a sitting position by him or herself	28	1	0	52.3	72	1	9	57.3	0.16
Picks up food and eats it	24	1	4	56.6	69	3	10	55.8	0.85
Pulls up to standing	25	4	0	52.0	65	1	16	57.4	0.26
Plays games like “peekaboo” or “patacake”	24	3	2	55.0	67	2	13	56.4	0.76
Calls you “mama” or “dada” or similar name	26	2	1	53.3	69	0	13	57.0	0.39
Looks around when you say things like “Where’s your bottle?“ or “Where’s your blanket?“	24	4	1	52.8	64	0	18	57.1	0.38
Copies sounds that you make	26	1	2	52.9	67	9	6	57.1	0.35
Walks across a room without help	29	0	0	53.5	77	0	5	56.9	0.18
Follows directions like “Come here” or “give me the ball”	28	1	0	51.8	71	4	7	57.5	0.13
Runs	29	0	0	53.5	77	0	5	56.9	0.18
Walks upstairs with help	28	0	1	55.4	78	0	4	56.2	0.75
Kicks a ball	27	2	0	57.8	80	0	2	55.4	0.29
Names at least 5 familiar objects like ball or milk	28	0	1	54.9	77	1	4	56.4	0.59
Names at least 5 body parts like nose, hand, or tummy	29	0	0	55.5	81	0	1	56.2	0.55
Climbs up a ladder at a playground	28	1	0	55.3	78	0	4	56.2	0.72
Uses words like “me” or “mine”	28	1	0	55.9	79	0	3	56.0	0.93
Jumps off the ground with two feet	28	1	0	55.9	79	0	3	56.0	0.93
Puts 2 or more words together like “more water” or “go outside”	29	0	0	55.0	80	1	1	56.3	0.40
Uses words to ask for help	28	1	0	55.4	78	1	3	56.2	0.73

^a^ Mann-Whitney U test

Out of the full MERG pediatric cohort (*N* = 608) there were 80 losses to follow-up, of whom 19 had microcephaly, 58 were offspring of the MERG Pregnant Women Cohort, and 3 were from the control group of the MERG case-control Study. To analyze if the 80 losses would result in bias, we compared the characteristics of children lost to and included in follow-up. For children with microcephaly, both groups were similar. Of the 58 children born to mothers from the Pregnant Women Cohort, 16 were born to mothers who were PCR- negative for ZIKV and did not meet the inclusion criteria for the current study; we therefore excluded them from the comparison (Table 4). From the remaining 42 children considered lost to follow-up, 31 mothers were not tested by PCR during pregnancy and 11 were PCR + for ZIKV during pregnancy.

**Table 4:** – Comparison of the children with Zika related microcephaly and children exposed without microcephaly, included in the analysis versus losses to follow-up, in relation to biological, clinical and radiological characteristics

Biological, clinical and radiological characteristics		Microcephaly		Children born to PCR + mothers	
		Losses (*n* = 19)	Included (*n* = 134)	Losses* (*n* = 42)	Included (*n* = 94)
Sex, N (%)	Female	7 (42.1)	24 (41.4)	19 (45.2)	47 (50.0)
	Male	11 (57.9)	34 (58.6)	23 (54,8)	47 (50.0)
Prematurity, Weeks of Gestational Age, N (%)	< 37	2 (20.0)	20 (15,0)	4 (22.2)	6 (7.9)
	≥ 37	3 (60.0)	109 (85,0)	14 (77.8)	70 (92.1)
	Missing	14	5	24	18
Birthweight, g, N(%)	< 2500	2 (25.0)	38 (29.9)	4 (15.4)	5 (7.1)
	2500–2999	3 (37.5)	50 (39.4)	8 (30.8)	14 (20.0)
	≥ 3000	3 (37.5)	39 (30.7)	14 (53.8)	51 (72.9)
	Missing	11	7	16	24
Mother’s age	Years		25.5 (22–34)	28 (21–31)	25 (20–32)
Image abnormalities	Yes	2 (33.6)	109 (89.3)	3 (23.1)	5 (11.6)
	No	4 (66.7)	13 (10.7)	13 (76.9)	38 (88.4)
	Missing	13	12	26	51

*11 children born to PCR + mothers plus 31 children whose mothers were not tested PCR

## Discussion

Prior studies have highlighted both the urgent need for further investigation of neurodevelopmental outcomes in children with prenatal ZIKV exposure and the challenge of identifying instruments appropriated for evaluating children across the spectrum of CZS, especially those with severe microcephaly  [17–19].

In this investigation, we applied the SWYC screening test to assess the neurodevelopment and behavior of prenatally ZIKV-exposed children with and without microcephaly who were born during the ZIKV microcephaly epidemic (2015–2017) in Pernambuco in the Northeast of Brazil [20].

According to the SWYC screening, virtually all participants with severe microcephaly (Group 1) and approximately two-thirds of participants with moderate microcephaly (Group 2) were considered ‘at risk of development delay.’ In comparison, 13.8% of ZIKV-exposed normocephalic children (Group 3) and 21.7% of control group children (Group 4) were identified by SWYC assessment as being ‘at risk.’.

The high frequency of ‘risk of development delay’ observed in children with microcephaly is likely attributable to the severity of the cerebral damage. Cerebral malformations generally indicate a poor prognosis in terms of neurodevelopmental function [21]. In a child with microcephaly caused by etiologies other than ZIKV, the risk of intellectual disability has been estimated to be 10.5% for HC between − 2 and − 3 SD, 51.2% for HC between − 3 and − 4 SD, and 100% for HC below − 4SD [22].

In this cohort of children with ZIKV-related microcephaly, the majority of cases in Group 1 had marked chronic encephalopathy and extensive intraparenchymal cortical calcifications, among other neuroimaging abnormalities. Furthermore, the frequency of central nervous system malformations, pyramidal syndrome, epilepsy, inadequate response to visual and auditory stimuli were higher in Group 1 than in the other groups evaluated. These neuroimaging and clinical findings are predictors of severe neuropsychomotor impairment and are among the phenotypic characteristics of CZS [1, 2, 23–25].

Consistent with our findings, a 2019 Brazilian investigation assessing children with cerebral palsy and probable CZS, of whom 97.5% had microcephaly, using the Bayley Scale of Infant and Toddler Development III (Bayley-III) reported scores below 70 (i.e., suggesting severe developmental delays) for almost all participants across all three scales: cognitive, 95.1%; language, 97.6%; motor, 97.6% [23].

A case series study assessing 24 children with ZIKV-related microcephaly in Northeast Brazil using the Denver Developmental Screening Test II also found a high degree of impairment for neuropsychomotor development. The study reported that children with a mean age of 19.9 months scored, on average, development milestones equivalent of ages 2.1 to 3.4 months, across the domains of language, motor, and personal/social skills [26].

Microcephaly, of any severity, is considered a useful indicator for developmental delays. However, in our study we compared neurodevelopment in children with severe and moderate microcephaly and observed, that among children with moderate microcephaly, 35% “Appear to Meet Age Expectations” using the SWYC assessment. Therefore, this strategy of classifying the microcephaly into moderate or severe allowed us to observe that the predictive value of SWYC varies according to the severity of microcephaly.

Although the frequency of cases with moderate microcephaly in our sample ‘at risk of developmental delay’ was lower than the frequency in cases with severe microcephaly, the percentage of ‘at risk’ children was higher than that found in the normocephalic groups, which included the ZIKV-exposed and control children. Indeed, both normocephalic groups had similar frequencies of ‘at risk’ children to each other and to the percentage of ‘at risk’ children in the general population that would be expected to be found with screening tests [27].

Prior to this study, few studies with comparable methods have investigated the development of children without microcephaly who were exposed to ZIKV prenatally. In a cohort in the Southeast Brazil that was assessed using Bayley-III, 28% of ZIKV-exposed children presented with at least one below average score (i.e., scores < 85 − 70) for cognitive, language, and motor function [28]. In using a screening test instead of a more comprehensive developmental assessment, such as Bayley-III, we would expect an even higher percentage of children to be identified as being at risk of developmental delay; however, our results from the SWYC screening suggest a lower frequency of children at risk of developmental delay in this cohort than compared with the Rio de Janeiro sample. Nevertheless, we note that a normal SWYC test cannot exclude subsequent later-onset neurodevelopment repercussions. Therefore, we recommend that children with prenatal ZIKV exposure should undergo a longitudinal evaluation, using additional and more accurate and comprehensive tests, such as the Bayley-III [29].

Using the adapted SWYC form, the expected score would be the one predicted by the child’s age at assessment (i.e., the child should perform most of the milestones expected for their age, as well as the milestones of the lower age groups). This adaptation made it possible to observe that children with severe microcephaly were severely limited in their ability to achieve developmental milestones that were appropriate for their chronological age. Even though over 74% of the children assessed were > 24 months of age, over 80% were unable to perform tasks corresponding to the expected skill acquisition for 5–8 month of age, such as item 14 of the adapted form (“passes a toy from one hand to another”).

When children were divided into two age groups (14–24 months vs. 25–32 months), we observed no differences in the achievement of developmental milestones, with the exception of the item “Laughs”. The acquisition of this milestone by the older children may be explained by the fact that this is a predominantly socio-emotional skill, and less dependent on motor and cognitive functions, which are usually highly affected in these children. When comparing the performance of the older and younger groups, these findings suggest a significant limitation in the ability of the children with severe microcephaly to achieve new milestones as they get older, which may be explained by the severity of their neurological impairment.

Although the SWYC tool was not specifically designed to provide the deficit profile of development, this study demonstrates an additional application of the SWYC, which addresses the current lack of specific instruments for evaluating development in children with severe neurological impairment. Further follow-up studies and repeated measures will be valuable for confirming the observation that children with severe ZIKV-related microcephaly maintain the neurodevelopment far below their expected age.

Using the SWYC checklists, the risk for behavioral and emotional symptoms was observed to be similar among the groups. It is plausible that the SWYC questions related to behavior and emotional symptoms may have generated inconsistent responses for children with severe microcephaly, due to the children’s serious motor and intellectual limitations. Questions such as “Is your child interested in playing with other children”, “Does your child break things on purpose”, or “Is your child fidgety or unable to sit still” are likely out of context for most children with severe microcephaly. Therefore, we suggest that these results do not mean that Group 1 has lower risk for behavioral problems, but rather that children with severe microcephaly do not possess the cognitive, emotional and motor skills required to demonstrate the ‘at risk’ behaviors. Therefore, this result should be interpreted with caution.

Initial descriptions of CZS mentioned irritability as a frequent clinical finding [30]. Although this characteristic was often reported and observed in newborns and young infants with a phenotype typical for CZS, irritability became less evident as the children grew (personal observation of the authors). In this investigation, irritability was not a predominant complaint in Group 1, which may be related to the fact that most of these children were assessed after the second year of life, at which time irritability may have been less likely to be presented. It is not possible to determine whether the condition was resolved or if this behavior was modified by the frequent use of anticonvulsants in this population [31].

Also unexpectedly, children without microcephaly (Groups 3 and 4) demonstrated high frequencies of risk signs of behavioral and emotional symptoms (42.5% and 63.7%, respectively). Although the underlying cause in this cohort is currently unknown, we note that studies have shown a progressive increase of the prevalence of behavioral abnormalities in childhood worldwide [32–34].

## Limitations and strengths

Since cases of microcephaly were identified during an emerging epidemic, and since it is not possible to ensure that microcephaly was representative of all cases born during the Zika outbreak, there is a potential for selection bias. Specifically, the moderate cases of microcephaly that are less readily clinically detectable could be underrepresented in this cohort. In addition, a survival bias related to CZS severity cannot be excluded as the neurodevelopment assessments were performed at different ages across the clinical groups. Additionally, selection bias in group 3 may have occurred as 31 children without microcephaly and unknown prenatal ZIKV exposure status from the MERG pregnancy cohort were lost to follow-up and more likely than the included children to be born with low birth weight or prematurely, two characteristics associated with both ZIKV exposure and neurodevelopmental delay.

Nevertheless, efforts were undertaken to mitigate the potential for bias in this study. First, the field workers were trained to uniformly apply the SWYC. For normocephalic children, the staff were blinded to information about mothers’ gestational and ZIKV testing history; however, for children with microcephaly (Groups 1 and 2), a blinded assessment was not possible. Second, this study utilizes a validated translation of SWYC to minimize risks of cross-cultural biases and to achieve reliable and comparable measures of the developmental and behavioral domains.

The sample size was large enough to detect the differences among groups in relation to development milestones. For the behavioral evaluations, as the difference in the frequency of “suspected behavior abnormalities” between groups was not large, we found a low power of the study to detect differences of the magnitude. Power of comparison between groups 1 + 2 vs. group 3 = 31%; power of comparison between groups 1 + 2 vs. group 4 = 24%; power of comparison between groups 3 vs. group 4 = 63%.

Among the strengths of this study, we can highlight the possibility of comparing children with different levels of ZIKV involvement, including children with and without microcephaly, in addition to a control group. Also, the use of the adapted form not only enabled the detection of the delay, but also enabled us to define skills that were expected but not achieved, which allows earlier and more targeted multidisciplinary intervention to address the identified needs in children with severe microcephaly.

## Conclusions

Children with prenatal exposure to ZIKV may manifest different levels of neurodevelopmental impairment, and even amongst children with microcephaly, there is a variability in functional performance. Our results suggest that children with severe microcephaly do not seem to acquire new skills beyond a certain stage of development. To confirm these findings, we suggest that children with severe microcephaly should be evaluated with repeated measurements from the adapted form. For children with prenatal exposure to ZIKV at risk of developmental delay, we suggest repeated neurodevelopmental assessments using more accurate and comprehensive instruments, such as the Bayley-III. SWYC may be adopted as a screening tool, thereby enabling at-risk children to be referred for further detailed assessment and multidisciplinary care.

## Data Availability

The datasets used and/or analysed during the current study are available from the corresponding author on reasonable request.

## Abbreviations

BPSC: Baby pediatric symptom checklist
CT: Computed tomography
CZS: Congenital zika syndrome
HC: Head circunference
MERG: Microcephaly Epidemic Research Group
MRI: Magnetic resonance imaging
PCR: Polymerase chain reaction
PPSC: Preschool pediatric symptom checklist
RT-PCR: Reverse-transcription polymerase chain reaction
SD: Standard deviations
USG: Ultrasonography
SWYC: Survey of wellbeing of young children
ZIKV: Zika Virus
